# Expression of MiRNA-29b and MiRNA-31 and their diagnostic and prognostic values in Egyptian females with breast cancer

**DOI:** 10.1016/j.ncrna.2022.09.003

**Published:** 2022-09-06

**Authors:** Mona Ahmed Abbas, Ibrahim El Tantawy El Sayed, Azza Mohamed Kamel Abdu-Allah, Abul Kalam, Abdullah G. Al-Sehemi, Omar A. Al-Hartomy, Maha Salah Abd El-rahman

**Affiliations:** a Department of Medical Biochemistry and Molecular Biology, Faculty of Medicine, Menoufia, Egypt; b Department of Chemistry, Faculty of Science, Menoufia University, Egypt; c Medical Laboratory Department, College of Applied Medical Sciences, Taibah University, Yanbu, Saudi Arabia; d Research Center for Advanced Materials Science (RCAMS), King Khalid University, P.O. Box 9004, Abha, 61413, Saudi Arabia; e Department of Chemistry, College of Science, King Khalid University, P.O. Box 9004, Abha, 61413, Saudi Arabia; f Department of Physics, Faculty of Science, King Abdulaziz University, Jeddah, 21589, Saudi Arabia

**Keywords:** microRNA, Breast cancer, Biomarker, qRT-PCR, Expression

## Abstract

Breast cancer is a major health threat to women globally. Many circulating microRNAs are non-invasive cancer biomarkers. In this study, the expression of miR-29b and miR-31 was assessed in blood samples from 200 patients with breast cancer and wholesome volunteer women using quantitative reverse transcriptase PCR to evaluate their role in the disease. MiR-29b was significantly overexpressed in patients compared to controls. Multivariate regression analysis showed that it was an established risk factor for relapse and mortality. MiR-31 was significantly under-expressed in patients. It was an established risk factor for relapse and was strongly associated with mortality. For the prediction of relapse, miR-29b had a sensitivity of 81.25% and a specificity of 88.24% at a cutoff of > 30.09, while miR-31 had a sensitivity of 87.50% and a specificity of 79.41% at a cutoff of 0.12. The specificity was enhanced to 100% by combining the values of miR-29b and miR-31. In predicting mortality, miR-29b exhibited a sensitivity of 90% and a specificity of 97.5% at a cutoff of > 48.10. At a cutoff of 0.119, miR-31 exhibited a sensitivity of 87.50% and a specificity of 79.41%. High miR-29b expression and low miR-31 expression were linked with a low survival rate. MiR-29b and miR-31 could be useful markers for predicting breast cancer relapse and mortality.

## 1 Introduction

Breast cancer (BC) is the 2nd most common cancer globally [1]. In 2020, it was responsible for approximately 24.5% of all cancer cases and 15.5% of cancer deaths among women, placing it first in terms of incidence and mortality in most countries [2]. Every year, around 1.7 million new cases are detected globally, representing 25% of all newly diagnosed cancers among women [3]. Recent guidelines have advocated a classification of BC based on five molecular subtypes: luminal A, luminal B, luminal B HER2-positive, HER2-enriched, and triple-negative (basal) [4]. The most common subtype is luminal A, distinguished by the expression of estrogen receptor (ER), progesterone receptor (PR), Bcl-2, and the absence of Her2. It accounts for 50–60% of all breast cancer cases [5]. The luminal B subtype is distinguished by the presence of ER, PR, and the absence of Her2 [6]. These two tumor subtypes are linked to a better prognosis. Her2 positive subtypes account for 15–20% of all breast cancers. It is distinguished by high Her2 gene expression and a high proliferation rate. Basal-like breast cancer, which accounts for 10–20% of all breast carcinomas and is often associated with a poor prognosis, does not express any of the three markers (ER, PR, and Her2) [7].

MicroRNAs are short non-coding RNAs, 17–25 base pairs in length [8]. They influence genes post-transcriptionally via binding to the 3′ or 5′ non-translated sequences of selected messenger RNA (mRNA), preventing mRNA degradation or translation inhibition. In addition to their inhibitory action, miRNAs have been shown to promote increases in transcript levels, enhancing gene expression in certain circumstances [9]. They are regarded as master gene regulators because they impact all cancer-related characteristics, including cancer cell proliferation, apoptosis, tumorigenesis, and metastasis, acting as oncogenic or tumor suppressor miRNAs [10,11]. There is evidence that miRNAs contribute to the formation and propagation of breast cancer so that they can be used as diagnostic tools, predictive indicators, and therapeutic targets [12].

An altered miRNA expression profile can distinguish between cancer and healthy samples and classify specific molecular subtypes of BC based on a unique miRNA expression pattern associated with each subtype [7,13]. For example, in a meta-analysis of independent studies, van Schooneveld et al. defined specific miRNAs for each intrinsic subtype of BC [14].

MiR-29b is a part of the miR-29 group of microRNAs; it regulates a wide variety of cellular activities via targeting different mRNAs [15]. The miR-29b family members include miR-29b-1, found on chromosome 7q32.3 and miR-29b-2 on 1q32.2 [16]. The miR-29 family has been identified to be upregulated in various malignancies. The miR-29 family's aberrant expression is linked to carcinogenesis and cancer progression [17].

Increased expression of miR-29b inhibits apoptosis and suppresses the tumor suppressor phosphatase and tensin homolog (PTEN), allowing tumor cells to invade and migrate more efficiently [18]. MiR-31 is located at 9p21.3 near the p16-Arf-p15 locus; a genomic region deleted mostly in diverse cancer types [19]. MiR-31 is a tissue-specific miRNA with tumor-suppressing effects in some tissues while oncogenic effects in others [20]. It plays a crucial role in various malignancies, such as breast cancer, ovarian can-cer, lung cancer, colon cancer, and melanoma. MiR-31 is shown to have anti-metastatic properties in breast cancer [21,22].

This study aimed to profile miR-29b and miR-31 expressions in breast cancer patients to evaluate their role in the disease.

## 2 Materials and methods

### 2.1 Study participants and protocol

This case-control research enrolled 100 women diagnosed with breast cancer and 100 healthy women of comparable age who served as controls. Patients were chosen from Menoufia University's Clinical Oncology and Nuclear Medicine Department. Inclusion criteria were female gender, any histopathological subtype, and any stage of breast cancer. Patients with cardiac failure, kidney or liver diseases, and other malignancies were rejected. This research was authorized by the Menoufia University Faculty of Medicine's ethical committee.

All participants underwent the following: 1. Obtaining a thorough medical history. 2. General clinical examination. Patients were subjected to: 1. Bilateral breast ultrasonography. 2. Staging workup (chest X-ray, pelvic and abdominal ultrasonography in initial stages), chest computed tomography, abdominal and pelvic contrast studies, and bone scan or PET/CT scan in late stages). 3. Tumor staging was based on tumor node metastasis (TNM) categorization and grading on the Nottingham modification of the Bloom-Richardson system [23]. 4. Using the estrogen receptor (ER), progesterone receptor (PR), and Her2/neu status, breast cancer was classified into molecular subtypes. 5. With a two-year surveillance period, the Kaplan-Meier method was employed to assess survival [24]. 6. The Eastern Cooperative Oncology Group's (ECOG) performance status was determined through the Eastern Cooperative Oncology Group [25].

### 2.2 Specimen collection and laboratory investigations

Four milliliters of blood were sampled from each participant, put in a plain tube, left to coagulate for half an hour, centrifuged for 10 min at 4000 rpm, then 100 μl of the obtained fresh serum was used for total RNA extraction including microRNA and the rest was preserved at −80 °C to measure serum carbohydrate antigen 15–3 (CA15-3), carcinoembryonic antigen (CEA) by enzyme-linked immunosorbent assay (ELISA) technique using kits from Chemux BioScience, Inc., (USA), serum urea, creatinine, AST and ALT.

### 2.3 Quantification of serum miR-29b and miR-31 gene expression

Purifying miRNA from the serum was performed as specified by the manufacturer using the miRNeasy® Kits (Qiagen, Germany). To ensure the purity of the collected RNAs, a NanoDrop spectrophotometer (Thermo Scientific, USA) was used.

Following purification, the isolated miRNA was kept at −80 °C. The isolated miRNA was reverse transcribed into single-stranded complementary DNA (cDNA), utilizing the miScript II RT Kit (Qiagen, Germany). The reaction was carried out on ice in a total volume of 20 μl, which included 4 μl miScript HiSpec RT buffer, 2 μl miScript Nucleics Mix, 2 μl miScript™ reverse transcriptases, 2 μl nuclease-free H_2_O, and 10 μl isolated miRNA. The reaction was preceded by one cycle of 37 °C for 60 min, followed by 95 °C for 5 min in a 2720 Applied Biosystems thermal cycler (Singapore) to block the reverse transcriptase enzyme. The cDNA was stored at a temperature of −20 °C until the real-time PCR stage. Real-time PCR was carried out utilizing a miScript SYBR Green PCR kit (Qiagen, Germany). Prior to assay preparation, cDNA was diluted 1:5 with nuclease-free H_2_O in a net volume of 25 μl (12.5 μl SYBR Green Master Mix, 3.5 μl nuclease-free water, 4 μl diluted cDNA, 2.5 μl miScript universal primer, and 2.5 μl miScript primer assay). As the reference miRNA, RNU6 was used. The primers for mature miR-29b, −31, and RNU6 were supplied by Qiagen, Germany, and listed in Table 1. Samples were analyzed by an ABI 7500 real-time PCR instrument (software V.2.0.1) with cycling settings as: a 15-min initial phase at 95 °C, then three stages of 40 cycles for 15 s at 94 °C, 30 s at 55 °C, and 30 s at 70 °C. The relative expression levels of miRNAs were measured by the comparative cycle threshold (Ct) method relative to RNU6 snRNA. It was deduced from the 2^−ΔΔCt^ equation where ΔΔCt = (Ct miR-29b/-31 − Ct RNU6) _patients_ − (Ct miR-29b/-31 − Ct RNU6) _controls_.

**Table 1:** Sequences of miRNA primers used in real-time PCR.

MiR-29b	*Fowrward* 5′-GCTGGTTTCATATGGTGG-3′ *Reverse* 5′-GAACATGTCTGCGTATCTC-3′
MiR-31	*Fowrward* 5′-GCAAGATGCTGGCATAG -3′ *Reverse* 5′-GAACATGTCTGCGTATCTC-3′
RNU6	*Fowrward* 5′-CTCGCTTCGGCAGCACAT-3′ *Reverse* 5′-TTTGCGTGTCATCCTTGCG-3′

### 2.4 Statistical analysis

The dataset was loaded onto a computer and analyzed using IBM SPSS version 20.0 software. (IBM Corporation, Armonk, New York). The chi-square test was used to study group comparisons for categorical variables (Fisher or Monte Carlo). For normally distributed quantitative variables, the student's t-test was employed. The Mann–Whitney test was utilized for abnormally distributed quantitative variables. For abnormally distributed quantitative data, the Kruskal Wallis was used. The association between quantitative variables was investigated using Spearman's coefficient. The receiver operating characteristic curve (ROC) was employed to analyze the markers' value in predicting relapse or mortality. Regression was used to detect independent factors affecting relapse and mortality. Kaplan-Meier survival analysis with a log-rank test was used, and cox regression was done for the significant relationship between progression-free survival and overall survival. The reported results were statistically significant at the 5% level.

## 3 Results

### 3.1 The study population's fundamental characteristics

A sample of 200 women, 100 breast cancer patients (mean age 48.50 ± 10.98), and 100 age-matched healthy controls participated in this work. The demographic data and the laboratory parameters of the studied participants is shown in Table 2, and the distribution of breast cancer patients based on various aspects is shown in Table 3.

**Table 2:** The demographic data and the laboratory parameters of the studied participants.

	Case (n = 100)	Control (n = 100)	Test of Sig.	p
**Age (years)**				
Mean ± SD.	48.50 ± 10.98	48.72 ± 10.92	t = 0.142	0.887
**Marital status**				
Single	6 (6.0%)	3 (3.0%)	χ^2^ = 4.012	^MC^p = 0.271
Married	88 (88.0%)	90 (90.0%)		
Divorced	0 (0.0%)	3 (3.0%)		
Widow	6 (6.0%)	4 (4.0%)		
**Parity**				
Nullipara	6 (6.0%)	2 (3.0%)	χ^2^ = 2.083	^FE^p = 0.279
Para	94 (94.0%)	98 (98.0%)		
**Menstrual status**				
Premenopausal	62 (62.0%)	58 (58.0%)	χ^2^ = 0.333	0.564
Postmenopausal	38 (38.0%)	42 (42.0%)		
**Family history**				
Negative	92 (92.0%)	97 (97.0%)	χ^2^ = 2.405	^FE^p = 0.121
Positive	8 (8.0%)	3(3.0%)		
**BMI (Kg/m**^**2**^**)**				
Mean ± SD.	30.76 ± 4.87	29.79 ± 4.51	t = 1.470	0.143
**ALT (IU/L)**				
Mean ± SD.	27.0 ± 8.07	25.06 ± 4.33	U = 4754	0.539
**AST (IU/L)**				
Mean ± SD.	27.62 ± 6.33	26.50 ± 3.64	t = 1.534	0.127
**Urea (mg/dl)**				
Mean ± SD.	28.42 ± 6.60	27.07 ± 5.30	t = 1.595	0.112
**Creatinine (mg/dl)**				
Mean ± SD.	0.91 ± 0.38	0.79 ± 0.26	U = 4282.0	0.077

SD: Standard deviation, t: Student t-test, χ2: Chi square test, MC: Monte Carlo, FE: Fisher Exact, p: p value for comparing between the studied groups, ∗: Statistically significant at p ≤ 0.05, BMI: body mass index, ALT: alanine transaminase, AST: aspartate transaminase.

**Table 3:** The distribution of the enrolled breast cancer patients according to various parameters (n = 100).

Parameter	No. (%)
**Performance status ECOG**	
0	88 (88.0%)
1	12 (12.0%)
**Comorbidities**	
No	68 (68.0%)
DM	8 (8.0%)
HTN	8 (8.0%)
Hepatic	6 (6.0%)
Multiple	10 (10.0%)
**Pathological Subtype**	
IDC	92 (92.0%)
ILC	4 (4.0%)
Other	4 (4.0%)
**Pathological Stage**	
Stage I	8 (8.0%)
Stage II	36 (36.0%)
Stage III	40 (40.0%)
Stage IV	16 (16.0%)
**Metastasis status**	
No	76 (76.0%)
Yes	24 (24.0%)
**Mets Type**	
Un-applicable	76 (76.0%)
Oligometastasis (1–3)	8 (8.0%)
Widespread	16 (16.0%)
**Grade**	
Grade I	2 (2.0%)
Grade II	84 (84.0%)
Grade III	14 (14.0%)
**PT status**	
T1	14 (14.0%)
T2	48 (48.0%)
T3	26 (26.0%)
T4	12 (12.0%)
**PN status**	
N0	24 (24.0%)
N1	48 (48.0%)
N2	14 (14.0%)
N3	14 (14.0%)
**ER**	80(80.0%)
**PR**	76(76.0%)
**HER2 neu**	34(34.0%)
**Molecular subtype**	
Luminal A	22 (22.0%)
Luminal B1	40 (40.0%)
Luminal B2	18 (18.0%)
HER2 overexpressed	16 (16.0%)
Basal (triple negative)	4 (4.0%)
**Relapse or progression status**	
No relapse	68 (68.0%)
Relapse	32 (32.0%)
**Mortality**	
Survived	80 (80.0%)
Died	20 (20.0%)

### 3.2 The relations between expression profiles and clinical and laboratory parameters

**Fig. 1:**
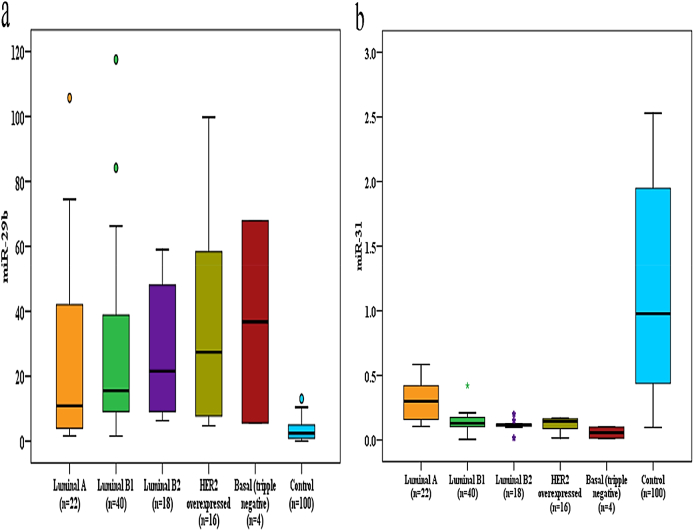
Fold changes in miR-29b (**a**) and miR-31 (**b**) in breast cancer subtypes compared to controls.

Regarding miR-29b expression, it was significantly overexpressed in breast cancer patients compared to controls (p < 0.001). Its high expression level was significantly related to the invasive lobular carcinoma (ILC) (p = 0.004), stage IV (p = 0.002), the presence of metastasis (p < 0.001), widespread metastasis (p < 0.001), the pathological node staging N3 (p < 0.001), relapsed cases (p < 0.001), died cases (p < 0.001), and cases with elevated CA15-3 levels (p = 0.039). Regarding miR-31 expression, it was significantly under-expressed in breast cancer patients compared to controls (p < 0.001). Low expression was significantly related to ECOG performance status 1 (p = 0.036), diabetic patients (p = 0.004), ILC (p = 0.004), stage IV (p = 0.001), the presence of metastasis (p < 0.001), widespread metastasis (p < 0.001), the tumor grade III (p = 0.041), the pathological tumor status T3 (p < 0.001), the pathological node status N3 (p < 0.001), the triple-negative subtypes (p < 0.001), relapsed cases (p < 0.001), died cases (p < 0.001), and cases with high CEA and CA15-3 levels (p < 0.001), as shown in (Table 4, Table 5 and Fig. 1 a & b).

**Table 4:** Comparison between breast cancer patients and control groups regarding miR-29b, miR-31, CEA, and CA15-3 levels.

	Case (n = 100)	Control (n = 100)	Test of Sig.	P
**MiR-29b** relative expression				
Median (IQR)	15.76(7.49–42.07)	2.44 (0.91–4.95)	U = 920.0∗	<0.001∗
**MiR-31** relative expression				
Median (IQR)	0.14(0.11–0.18)	0.98 (0.44–1.95)	U = 1300.0∗	<0.001∗
**CEA (ng/ml)**				
Normal (<5)	52(52.0%)	100(100.0%)	χ^2^ = 63.158∗	<0.001∗
Elevated (≥5)	48(48.0%)	0(0.0%)		
Median (IQR)	4.55 (2.10–8.0)	2.20 (1.50–3.0)	U = 2310.0∗	<0.001∗
**CA15**–**3 (IU/ml)**				
Normal (<30)	46(46.0%)	100(100.0%)	χ^2^ = 73.973∗	<0.001∗
Elevated (≥30)	54 (54.0%)	0(0.0%)		
Median (IQR)	45.95 (26.80–78.0)	14.20 (11.80–21.30)	U = 906.0∗	<0.001∗

IQR: the interquartile range, c^2^: Chi square test, U: Mann Whitney test, p: p value for comparing between the studied groups, ∗: Statistically significant at p ≤ 0.05.

**Table 5:** The association of miR-29b and miR-31 expression with clinicopathological parameters in breast cancer patients (n = 100).

	N	MiR-29b		P	MiR-31		P
		Mean ± SD.	Test of Sig.		Mean ± SD.	Test of Sig.	
Marital status							
Single	6	15.52 ± 5.58	H = 0.117	0.943	0.13 ± 0.02	H = 5.659	0.059
Married	88	29.63 ± 28.80			0.16 ± 0.12		
Widow	6	39.73 ± 51.33			0.26 ± 0.13		
Parity							
Nullipara	6	26.04 ± 13.30	U = 236.0	0.504	0.14 ± 0.15	U = 223.0	0.392
Para	94	29.61 ± 30.47			0.17 ± 0.12		
Menstrual status							
Premenopausal	62	27.82 ± 30.51	U = 930.0	0.078	0.16 ± 0.12	U = 1104.0	0.599
Postmenopausal	38	31.96 ± 28.53			0.18 ± 0.13		
Family history							
Negative	92	30.89 ± 30.46	U = 268.0	0.204	0.17 ± 0.13	U = 302.0	0.402
Positive	8	12.16 ± 6.50			0.16 ± 0.05		
Performance status ECOG							
0	88	27.53 ± 26.98	U = 416.0	0.235	0.18 ± 0.13	U = 330.0∗	0.036∗
1	12	43.08 ± 44.12			0.10 ± 0.06		
Comorbidities							
No	68	26.05 ± 27.98	H = 7.969	0.093	0.18 ± 0.12	H = 15.338	0.004∗
DM	8	36.62 ± 32.90			0.10 ± 0.08		
HTN	8	16.33 ± 11.20			0.23 ± 0.09		
Hepatic	6	51.57 ± 49.46			0.14 ± 0.20		
Multiple	10	43.45 ± 27.23			0.13 ± 0.11		
Pathological Subtype							
IDC	92	25.76 ± 25.56	H = 11.183∗	0.004∗	0.18 ± 0.12	H = 11.167∗	0.004∗
ILC	4	100.86 ± 19.27			0.03 ± 0.03		
Other	4	41.55 ± 38.74			0.09 ± 0.08		
Pathological Stage							
Stage I	8	28.40 ± 29.18	H = 15.009∗	0.002∗	0.33 ± 0.26	H = 34.901∗	0.001∗
Stage II	36	23.28 ± 26.44			0.21 ± 0.10		
Stage III	40	23.57 ± 24.88			0.12 ± 0.04		
Stage IV	16	58.20 ± 33.40			0.11 ± 0.13		
Metastasis status							
No	76	20.19 ± 22.65	U = 264.0∗	<0.001∗	0.19 ± 0.12	U = 356.0∗	<0.001∗
Yes	24	58.55 ± 30.91			0.11 ± 0.11		
Mets Type							
Un-applicable	76	20.19 ± 22.65	H = 27.535∗	<0.001∗	0.19 ± 0.12	H = 20.492∗	<0.001∗
Oligometastasis(1–3)	8	59.24 ± 27.36			0.10 ± 0.07		
Widespread	16	58.20 ± 33.40			0.11 ± 0.13		
Grade							
Grade I	2	55.77 ± 70.66	H = 1.685	0.431	0.37 ± 0.08	H = 6.411∗	0.041∗
Grade II	84	27.24 ± 28.16			0.17 ± 0.13		
Grade III	14	38.52 ± 32.26			0.12 ± 0.06		
PT status							
T1	14	26.56 ± 23.71	H = 7.292	0.063	0.27 ± 0.21	H = 24.965∗	<0.001∗
T2	48	25.33 ± 25.43			0.19 ± 0.11		
T3	26	44.94 ± 38.44			0.09 ± 0.04		
T4	12	15.24 ± 16.69			0.14 ± 0.03		
PN status							
N0	24	31.60 ± 32.74	H = 17.811∗	<0.001∗	0.25 ± 0.19	H = 21.586∗	<0.001∗
N1	48	23.05 ± 23.68			0.17 ± 0.08		
N2	14	16.29 ± 17.66			0.12 ± 0.02		
N3	14	60.45 ± 33.14			0.07 ± 0.07		
ER							
Negative	20	34.82 ± 31.40	U = 700.0	0.389	0.14 ± 0.09	U = 683.0	0.313
Positive	80	28.04 ± 29.30			0.18 ± 0.13		
PR							
Negative	24	31.57 ± 29.60	U = 828.0	0.498	0.14 ± 0.08	U = 773.0	0.262
Positive	76	28.70 ± 29.89			0.18 ± 0.13		
HER2 neu							
Negative	66	28.55 ± 31.65	U = 924.0	0.150	0.19 ± 0.14	U = 0.915	0.132
Positive	34	31.02 ± 25.84			0.13 ± 0.07		
Molecular subtype							
Luminal A	22	25.88 ± 33.64	H = 3.568	0.468	0.31 ± 0.15	H = 33.759∗	<0.001∗
Luminal B1	40	28.36 ± 29.82			0.14 ± 0.08		
Luminal B2	18	28.08 ± 20.12			0.12 ± 0.05		
HER2 overexpressed	16	36.43 ± 33.40			0.12 ± 0.05		
Basal(triple negative)	4	36.75 ± 35.97			0.06 ± 0.05		
Progression status							
No relapse	68	17.26 ± 19.11	U = 372.0∗	<0.001∗	0.20 ± 0.12	U = 284.0∗	<0.001∗
Relapse	32	55.16 ± 31.91			0.10 ± 0.10		
Mortality							
Survived	80	17.93 ± 14.90	U = 116.0∗	<0.001∗	0.18 ± 0.12	U = 246.0∗	<0.001∗
Died	20	75.26 ± 30.05			0.10 ± 0.12		
CEA (ng/ml)							
Normal (<5)	52	22.98 ± 24.97	U = 984.0	0.069	0.21 ± 0.14	U = 576.0∗	<0.001∗
Elevated (≥5)	48	36.34 ± 32.95			0.12 ± 0.08		
CA15-3 (IU/ml)							
Normal (<30)	46	21.48 ± 24.47	U = 944.0∗	0.039∗	0.23 ± 0.14	U = 416.0∗	<0.001∗
Elevated (≥30)	54	36.13 ± 32.21			0.12 ± 0.08		

SD: Standard deviation, U: Mann Whitney test, H: H for Kruskal Wallis test, p: p value for comparing between different categories, ∗: Statistically significant at p ≤ 0.05.

### 3.3 The prognostic significance of miR-29b and miR-31 in breast cancer

By applying univariate logistic regression analysis, the pathological subtype (invasive ductal carcinoma (IDC)), the pathological stage of the tumor (≥3), presence of metastasis, tumor grade (III), pathological tumor status (≥3), pathological nodal status (≥3), and the expression level of miR-29b and miR-31 were revealed to have a significant association with relapse in breast cancer patients. The pathological subtype of the tumor (IDC), the presence of metastasis, pathological tumor status (≥3), pathological nodal status (≥3), and the expression level of miR-29b and miR-31 were noted to be significantly related to mortality in breast cancer patients, as shown in (Table 6).

**Table 6:** Univariate logistic regression analysis for the factors affecting relapse and mortality in breast cancer patients.

	Relapse		Mortality	
	P	OR (95%C.I)	P	OR (95%C.I)
**MiR-29b**	**<0.001∗**	0.0(0.0–0.0)	**0.006∗**	0.0(0.0–0.0)
**MiR-31**	**<0.001∗**	1.056(1.033–1.081)	**<0.001∗**	1.105(1.058–1.153)
**Age (years)**	**0.276**	1.017(0.986–1.049)	**0.362**	0.979(0.935–1.025)
**Parity (Para)**	**0.340**	0.446(0.085–2.344)	**0.999**	–
**Menstrual status (Postmenopausal)**	**0.711**	1.177(0.498–2.783)	**0.187**	0.475(0.157–1.434)
**Family history**	**0.999**	–	**0.999**	–
**BMI (Kg/m2)**	**0.059**	0.918(0.843–1.0)	**0.089**	0.921(0.838–1.013)
**Performance status ECOG**	**0.723**	1.209(0.424–3.448)	**0.277**	2.250(0.603–8.396)
**Pathological Subtype (IDC)**	**0.017∗**	0.131(0.025–0.693)	**0.001∗**	0.060(0.011–0.327)
**Pathological Stage (≥3)**	**<0.001∗**	10.0(3.155–31.696)	**0.164**	2.111(0.737–6.046)
**Mets**	**<0.001∗**	72.600(14.760–357.087)	**0.004∗**	4.714(1.651–13.461)
**Grade (III)**	**0.036∗**	3.444(1.081–10.973)	**0.392**	1.750(0.486–6.297)
**PT status (≥3)**	**0.035∗**	2.537(1.069–6.019)	**0.027∗**	3.115(1.135–8.550)
**PN status (≥3)**	**<0.001∗**	19.800(4.085–95.968)	**0.028∗**	3.857(1.158–12.850)
**ER**	**0.748**	0.844(0.300–2.372)	**0.217**	0.495(0.162–1.512)
**PR**	**0.733**	1.190(0.437–3.242)	**0.484**	0.677(0.228–2.017)
**HER2 neu**	**0.399**	1.531(0.640–3.667)	**0.673**	0.796(0.275–2.300)

OR: Odd's ratio, C.I: Confidence interval, LL: Lower limit, UL: Upper Limit, #: All variables with p < 0.05 was included in the multivariate, ∗: Statistically significant at p ≤ 0.05.

By applying multivariate logistic regression analysis, pathological stage of the tumor (≥3), presence of metastasis, miR-31 under expression, and miR-29b overexpression were independent risk factors for relapse. In comparison, only miR-29b overexpression was independently linked to higher mortality risk.

**Fig. 2:**
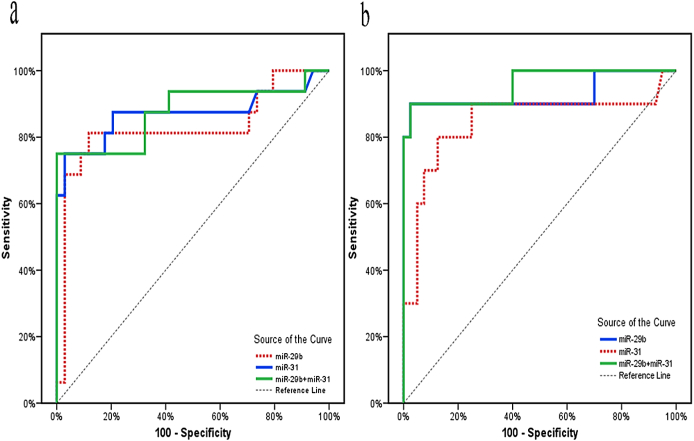
ROC curve for miR-29b and miR-31 for predicting relapse **(a)** and mortality **(b).**

The potential value of miR-29b and miR-31 in the prediction of relapse was evaluated via ROC curve analysis. MiR-29b had a sensitivity of 81.25%, a specificity of 88.24%, a 76.5% PPV, and a 90.9% NPV at a cutoff of > 30.09. At a cutoff of ≤ 0.12, miR-31 had a sensitivity of 81.25%, specificity of 79.41%, 66.7% PPV, and 93.1% NPV. The specificity was enhanced to 100% by combining the values of miR-29b and miR-31, as shown in (Table 7 & Fig. 2a).

**Table 7:** Validity of miR-29b and miR-31 as predictors of relapse in breast cancer patients.

	AUC	P	95% C.I	Cut off	Sensitivity	Specificity	PPV	NPV
**MiR-29b**	0.829	<0.001∗	0.725–0.933	>30.09	81.25	88.24	76.5	90.9
**MiR-31**	0.869	<0.001∗	0.773–0.966	≤0.12	81.25	79.41	66.7	93.1
**MiR-29b + MiR-31**	0.877	<0.001∗	0.789–0.965		75.0	100.0	100.0	89.47

AUC: Area Under a Curve, p value: Probability value, CI: Confidence Intervals, NPV: Negative predictive value, PPV: Positive predictive value, ∗: Statistically significant at p ≤ 0.05.

The utility of miR-29b and miR-31 in predicting mortality was also assessed using ROC curve analysis. MiR-29b exhibited a sensitivity of 90%, a specificity of 97.5%, a PPV of 90%, and an NPV of 97.5% when the cutoff was > 48.10. MiR-31 exhibited a sensitivity of 80%, specificity of 75%, 44.4% PPV, and 93.7% NPV at a cutoff of ≤ 0.119. Combining the values of miR-29b and miR-31 did not improve the results, as shown in (Table 8& Fig. 2b).

**Table 8:** Validity of miR-29b and miR-31 as predictors of mortality in breast cancer patients.

	AUC	P	95% C.I	Cut off	Sensitivity	Specificity	PPV	NPV
**MiR-29b**	0.927	<0.001∗	0.835–1.0	>48.10	90.0	97.5	90.0	97.5
**MiR-31**	0.846	0.001∗	0.723–0.970	≤0.119	80.0	75.0	44.4	93.7
**MiR-29b + MiR-31**	0.957	<0.001∗	0.903–1.0		80.0	97.50	88.89	95.12

AUC: Area under a Curve, p value: Probability value, CI: Confidence Intervals, NPV: Negative predictive value, PPV: Positive predictive value, ∗: Statistically significant at p ≤ 0.05.

**Fig. 3:**
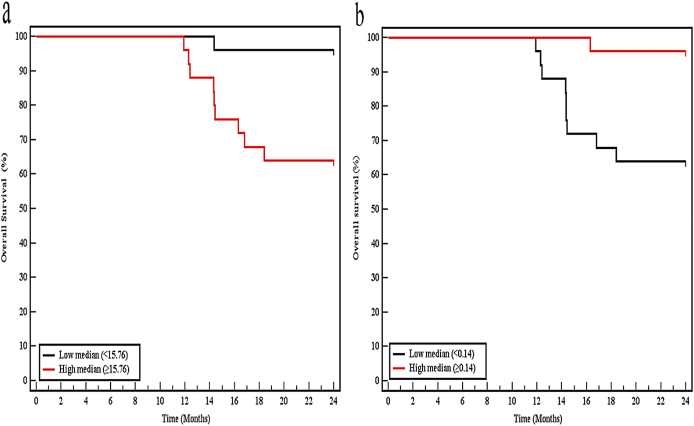
Kaplan-Meier survival curve for overall survival in breast cancer patients (n = 100) with miR-29b (**a**) and miR-31 (**b**).

To further assess the prognostic significance of miR-29b and miR-31 in breast cancer, we conducted a Kaplan-Meier survival analysis based on miR-29b and miR-31 expression levels and patient survival records. Patients were subdivided into high and low miRNA expressions using the median miRNA expression value as the cutoff. For miR-29b, the survival analysis demonstrated that by the end of the 2 years follow-up period the survival rate there was a highly significant statistical difference in the survival rate among patients with a low miR-29b expression (96%) and patients with higher expression levels (64%) (Log-rank p < 0.001). For miR-31, the survival analysis revealed that there was a highly significant statistical difference in the survival rate between patients with a low miR-31 expression (65.4%) and patients with higher expression levels (95.8%) (Log-rank p < 0.001), as shown in (Table 9, Fig. 3 a&b).

**Table 9:** Kaplan-Meier survival analysis for overall survival with miR-29b and miR-31 in breast cancer patients.

	Mean	95% C.I		% End of study	Log rank	
		LL	UL		χ2	p
**MiR-29b**						
Low median (<15.76)	23.613	23.09	24.14	96.0	15.954∗	<0.001∗
High median (≥15.76)	20.604	19.30	21.91	64.0		
**MiR-31**						
Low median (<0.14)	20.659	19.37	21.95	65.4	14.658∗	<0.001∗
High median (≥0.14)	23.659	23.24	21.11	95.8		

CI: Confidence Intervals, LL: lower limit, UL: upper limit.

## 4 Discussion

The prevalence of breast cancer has significantly expanded over recent years [26]. Breast cancer recurrence and metastasis remain critical clinical challenges, despite breakthroughs in the technologies used for diagnosis and therapy. As a result, identifying biomarkers or procedures for early discovery of malignancy or relapse after curative surgery utilizing minimally intrusive diagnostics is critical [27].

An increasing number of studies have focused on miRNAs as biomarkers in cancer diagnosis and treatment; however, the literature's disparate and contradictory findings are a major roadblock to clinical implementation [28]. In our research, we assessed the expression of miR-29b and miR-31 and their diagnostic and predictive values in patients with breast cancer.

In this study, miR-29b was significantly overexpressed in breast cancer patients compared to controls. Its high expression level was significantly related to the ILC, stage IV, the presence of metastasis, widespread metastasis, the pathological node staging N3, relapsed cases, died cases, and cases with elevated CA15-3 levels. This matches the findings of Shaker et al. [29], that miR-29b-2 expression correlated with nodal affection, tumor size, and metastasis stage. It was more expressed in advanced breast cancer cases, giving it the potential to be a diagnostic and prognostic marker. In contrast, Drago-Ferrante et al. [30] reported that miR-29b-1-5p was negatively regulated in human triple-negative breast cancer tissues and played a tumor-suppressive effect. According to Muluhngwi et al. [31], decreased expression of miR-29a-3p and miR-29b-3p has been linked to a lower overall BC survival rate. Li et al. [32] demonstrated that miR-29b expression is decreased and can operate as a tumor suppressor in cancer colon. In gastric cancer, Wang et al. [33] revealed down expression of miR-29b, which is linked to the matrix metalloproteinase 2 (MMP2) related cancer infiltration and dissemination. MiR-29a and miR-29b are negatively regulated in myeloid leukemia [34]. These variable findings were explained by Kwon [35], who reported that the miR-29 family members (miR-29a, miR-29b-1, miR-29b-2, and miR-29c can serve as oncogenic miRNAs or as tumor suppressor miRNAs according to the tissue.

Regarding cancer metastasis, miR-29b is well-known for having opposing effects depending on the cell, tumor type, or conditions [36]. In this study, we revealed that miR-29b is highly upregulated in the presence of widespread metastasis. This aligns with the results of Wang et al. [27], who found that metastatic breast cancer cells expressed more miR-29b than non-metastatic breast cancer cells and that miR-29b overexpression was linked to advanced cancer stage, lymph node involvement, and a poor outcome. They suggested that the possible mechanism is that overexpression of miR-29 decreases PTEN expression, thereby activating the PI3K pathway and increasing PI3K level, which recruits PDK1 and AKT to the cell membrane, enhancing cell growth, migration and metastasis. In contrast, the results of Chou et al. [37] showed that miR-29b inhibited metastasis in breast cancer. In carcinoma-associated fibroblasts (CAFs), miR-29b is dramatically negatively regulated, affecting chemokine secretion and its role in breast cancer cell division, therapy tolerance, and dissemination [38]. By targeting CCL11 and CXCL14, MiR-29b encore in CAFs decreases breast cancer cell survival and metastasis. In gastric cancer, miR-29b-3p was found to inhibit the emigration and colonization of cells by modulating the autophagy-related protein MAZ [39], while in colorectal cancer, overexpression of miR-29b suppresses the epithelial-mesenchymal transition and angiogenesis via disrupting the ETV4-dependent stimulation of the extracellular signal-regulated kinase (ERK) cascade [40]. In our study, miR-29b was noted to be an independent risk factor for relapse and mortality. Also, Shinden et al. [41] reported that miR-29b expression in primary breast cancer tumors was a reliable predictor of overall survival. Although Zhai et al. [42] demonstrated that a breast cancer patient's marital status can affect their survival; it was not a factor affecting the overall survival rate in our study.

In the current study, patients had considerably lower levels of miR-31 than controls. Its low expression level was found to be significantly related to ECOG performance status 1, diabetic patients, ILC, stage IV, the presence of metastasis, widespread metastasis, the tumor grade III, the pathological tumor status T3, the pathological node status N3, the triple-negative subtypes, relapsed cases, died cases and cases with high CEA and CA15-3 levels. This is backed by the conclusions of Luo et al. [43], who discovered that miR-31 was downregulated in triple-negative breast tumor tissues and stem cells. It is also downregulated in gastric [44], ovarian [45], and prostate cancers [46].

On the other hand, Lv et al. [47] investigated the involvement of miR-31 in modulating the activity of mammary stem cells and the development of breast cancer. They discovered that miR-31 was overexpressed in basal-like human BC, implying that miR-31 is crucial in mammary stem cell division and tumorigenesis via stimulation of the Wnt signaling system. In contrast, Niu et al. [48] found that miR-31 is highly expressed in breast tumors and mammary cells in vitro and that it can boost their growth and mammary epithelial multiplication; knocking out miR-31 repressed breast tumor progression, decreased breast cancer stem cell populations, cancer ability, and lung metastatic spread. It is also overexpressed in lung [49], colorectal [50], head and neck squamous cell [51], and esophageal squamous cell malignancies [52]. This varied up and downregulation of miR-31 in various malignancies was described by Yu et al. [53]; they reported that the proximity between miR-31 and the tumor suppressor gene cyclin-dependent kinase inhibitor 2A gene locus leads to their co-deletion or hypermethylation, resulting in reduced miR-31 expression in gastric, liver, breast, ovarian, and prostate malignancies. MiR-31, on the other hand, has oncogenic potential in other cancers, such as lung and colorectal cancers, because the KRAS can increase the level of miR-31 by stimulating its promoter. In our study, low expression of miR-31 was significantly linked to metastasis, especially the widespread type. This is consistent with the findings of Luo et al. [43], who found that miR-31 expression was negatively correlated with breast cancer metastasis. They discovered that miR-31 regulates WAVE3, a metastasis promoter protein that is required for epithelial to mesenchymal transition, an initial process in the invasion-metastasis sequence, and found an inverse relation between WAVE3 and miR-31 expression levels in invasive versus non-invasive breast cancer cells. Augoff et al. [54] state that miR-31 significantly contributes to BC progression and metastasis by regulating a group of pro-metastatic target genes such as WAVE3, RhoA, Radexin, and several integrin subunits that regulate vital steps in the invasion-metastasis cascade. These genes control essential steps in the invasion-metastasis process. Combined miR-29b and miR-31 measurement is a good predictor for relapse with a specificity of 100%.

## 5 Conclusions

A combined miR-29b and miR-31 expression measurement is a good predictor for relapse with a specificity of 100%. According to the previous results, miR-29b and miR-31 may be helpful diagnostic and prognostic markers for breast cancer. Dysregulation of miR-29b and miR-31 are programrisk factors for relapse and high mortality in breast cancer patients.

## Funding

The authors acknowledge the support and funding of 10.13039/501100007446King Khalid University through Research Center for Advanced Materials Science (RCAMS) under grant no: RCAMS/KKU/0010/21. Maha S. Abd El-rahman acknowledges the financial support of 10.13039/501100002349Academy of Scientific Research and Technology of “Next Generation Scientists” program.

## Institutional review board statement

The study was conducted according to the guidelines of the Declaration of Helsinki, and approved by the local Ethics Committee of Faculty of Medicine, Menoufia University (approval code: 5–2022 BIO 10–2).

## Informed consent statement

Written informed consent was obtained from all subjects involved in the study.

## Data availability statement

The data are available from the authors on reasonable request.

## CRediT authorship contribution statement

**Mona Ahmed Abbas:** Visualization, Investigation, Supervision, Writing – review & editing. **Ibrahim El Tantawy El Sayed:** Conceptualization, Methodology, Supervision, Writing – review & editing. **Azza Mohamed Kamel Abdu-Allah:** Data curation, Writing – original draft, Writing – review & editing. **Abul Kalam:** Validation. **Abdullah G. Al-Sehemi:** Visualization, Investigation. **Omar A. Al-Hartomy:** Methodology, Investigation. **Maha Salah Abd El-rahman:** Investigation, Writing – original draft.

## Declaration of competing interest

The authors declare no conflict of interest.
